# Reduction of Proton Pump Inhibitor Dependence in Gastroesophageal Reflux Disease Following Isometric Diaphragmatic Resistance Training With a Novel Respiratory Muscle Training Device: An Autobiographical Case Report

**DOI:** 10.7759/cureus.107172

**Published:** 2026-04-16

**Authors:** Gukbin Park

**Affiliations:** 1 Internal Medicine, Haeundae Central Internal Medicine Clinic, Busan, KOR

**Keywords:** crural diaphragm, diaphragmatic resistance training, gastroesophageal reflux disease, isometric breathing exercise, lower esophageal sphincter, ppi dependence, proton pump inhibitor, respiratory muscle training, self-experimentation, transient les relaxation

## Abstract

Gastroesophageal reflux disease (GERD) has been associated with transient lower esophageal sphincter relaxations (TLESRs), which are considered a predominant mechanism of reflux and are modulated in part by the crural diaphragm. Long-term proton pump inhibitor (PPI) therapy remains the standard of care but is associated with drug dependency and systemic adverse effects. Strengthening the crural diaphragm through resistive respiratory training represents a mechanistically plausible non-pharmacological approach. We report a case in which a 14-day isometric diaphragmatic resistance training protocol using a novel prototype device substantially reduced PPI requirement and GERD symptom burden in a patient with over a decade of PPI-dependent GERD.

A 38-year-old male physician with a more than 10-year history of GERD, refractory to multiple prior acid-suppressive agents and maintained on dexlansoprazole 60 mg daily, voluntarily discontinued his morning PPI. Immediate and consistent nocturnal symptoms, heartburn, regurgitation, and sleep disturbance, emerged upon discontinuation, necessitating nightly rescue dexlansoprazole use. Following a five-day informal observation period and two days of structured formal baseline recording (evening binary GerdQ score 4/6 on both days), a 14-day diaphragmatic resistance training protocol was initiated using a novel prototype respiratory muscle training device incorporating a safety algorithm to limit inspiratory negative pressure below the reflux induction threshold. The protocol comprised three sets of 10 repetitions daily with 2-second isometric inspiratory and expiratory holds and 3-minute inter-set rest periods. Over 14 days, the mean evening GerdQ score decreased to 1.1/6 (71% reduction from baseline), PRN dexlansoprazole was required on only 3 of 14 days (21%), and sleep disturbance was recorded on a single occasion (Day 3 only).

This case demonstrates that isometric diaphragmatic resistance training was associated with marked reduction in PPI requirement and GERD symptom burden in a patient with longstanding PPI-dependent disease. The findings support crural diaphragm strengthening as a viable non-pharmacological target in GERD management, warranting controlled trials with objective esophageal endpoints.

## Introduction

Gastroesophageal reflux disease (GERD) is among the most prevalent gastrointestinal disorders globally, affecting approximately 10-20% of the Western population and less than 5% in Asia [1]. The condition is characterized by the retrograde flow of gastric contents into the esophagus, producing heartburn and regurgitation that substantially impair quality of life, and predisposing to complications including Barrett’s esophagus and esophageal adenocarcinoma [2]. Among the mechanisms implicated in GERD pathophysiology, lower esophageal sphincter (LES) dysfunction, including reduced basal LES pressure, impaired esophageal peristaltic function, and transient LES relaxations (TLESRs), plays a central role in permitting the retrograde movement of gastric contents into the esophagus [3].

The crural diaphragm constitutes the extrinsic component of the esophagogastric junction barrier, encircling the LES at the esophageal hiatus. During inspiration, the chest cavity generates negative intrathoracic pressure, which, in the presence of a competent LES, does not result in reflux. However, in patients with a weakened LES, this inspiratory negative pressure can act as a suction force that draws gastric contents into the esophagus. The crural diaphragm serves as a critical safety mechanism against this phenomenon: its synchronous contraction during inspiration augments LES pressure, counteracting the pressure gradient and preventing reflux [4]. Impairment of this structure through hiatal hernia, obesity, or deconditioning compromises this protective mechanism and increases TLESR susceptibility.

Proton pump inhibitors (PPIs) are the pharmacological cornerstone of GERD management, but long-term use has been associated with a range of serious adverse events including cardiovascular disease, acute kidney injury, chronic kidney disease, dementia, pneumonia, gastric cancer, Clostridium difficile infection, and osteoporotic fractures, and nutrient deficiencies including vitamin B12, iron, and magnesium, as well as clinically significant drug dependency [5]. A subset of patients requires daily high-dose PPI to maintain symptom control, with immediate symptomatic relapse upon discontinuation. Non-pharmacological interventions targeting the underlying mechanical deficit are therefore of considerable clinical interest.

Eherer et al. demonstrated in a randomized controlled trial that structured diaphragmatic breathing exercises significantly reduced esophageal acid exposure and improved GERD symptom scores compared to sham training [6]. The mechanism is believed to involve progressive crural diaphragm strengthening, increased LES resting pressure, and reduced TLESR frequency. However, conventional breathing exercises are effort-dependent, lack standardized resistance loading, and do not permit progressive overload, a fundamental principle of musculoskeletal training necessary for sustained adaptation.

We developed a novel prototype respiratory muscle training device that delivers calibrated isometric resistance during both inspiratory and expiratory phases through a pressure-sensing active valve control mechanism, enabling reproducible and progressive loading of the respiratory musculature, including the crural diaphragm. In this report, we describe the first self-experimentation case using this device in a physician with a more than 10-year history of PPI-dependent GERD who had failed multiple prior acid-suppressive agents and was maintained on dexlansoprazole 60 mg daily. Following voluntary PPI discontinuation and a structured observation period, we document the effect of 14 days of isometric diaphragmatic resistance training on symptom burden and rescue PPI requirement.

## Case presentation

Patient background and PPI discontinuation phase

I am a 38-year-old board-certified internist and gastroenterologist with a clinical diagnosis of GERD established over a decade prior. Despite trials of multiple acid-suppressive agents, adequate symptom control was achieved only with dexlansoprazole 60 mg once daily in the morning, which I had taken continuously for an extended period. When maintained on this regimen, I remained entirely asymptomatic throughout the day and night.

Approximately five days prior to formal study initiation, I voluntarily discontinued morning dexlansoprazole. Upon discontinuation, nocturnal symptoms emerged immediately and consistently each evening: heartburn, regurgitation, and sleep disturbance, necessitating rescue dexlansoprazole intake on each occasion. Morning symptoms were absent throughout this period, consistent with the residual pharmacodynamic effect of the prior morning dose and the inherent diurnal pattern of gastric acid secretion. This five-day informal observation period confirmed both my PPI dependency and the reproducibility of the nocturnal symptom pattern.

Formal baseline recording

The two days immediately preceding exercise initiation (Day −2 and Day −1) were designated as the formal baseline period and documented using a binary-adapted GerdQ. Both days demonstrated identical evening symptom profiles: heartburn present, regurgitation present, sleep disturbance present, and rescue dexlansoprazole required (binary GerdQ score 4/6). Morning symptoms were absent on all assessment points throughout the entire study.

The consistently absent morning symptoms in the context of prominent nocturnal symptoms are mechanistically informative. In the upright position, gravitational forces assist esophageal clearance and reduce the likelihood of refluxate reaching the proximal esophagus. In the supine position, this gravitational defense is eliminated, rendering the esophagogastric junction entirely dependent on intrinsic LES competence and crural diaphragm tone for barrier function. The exclusively nocturnal symptom pattern in this case therefore strongly implicates TLESR-mediated reflux as the dominant mechanism, precisely the target of the training intervention. This mechanistic specificity lends particular credibility to this case as a model for evaluating crural diaphragm-directed therapy, noting that not all GERD presentations share this positional pattern.

Device description

The device used in this study is a prototype respiratory muscle training apparatus incorporating a microelectromechanical pressure sensor interfaced with a microcontroller for real-time pressure monitoring. The device operates by continuously monitoring intraluminal pressure during both inspiratory and expiratory phases and engaging an active valve mechanism when a predefined pressure threshold is reached, at which point airflow is restricted and the user is required to sustain isometric muscular effort against the fixed resistance. During the inspiratory phase, a dedicated safety algorithm caps the negative pressure ceiling below a literature-based estimated reflux induction threshold, with the aim of ensuring that diaphragmatic loading is achieved without generating a suction gradient sufficient to draw gastric contents into the esophagus. It should be noted that my individual reflux induction threshold was not directly measured by esophageal manometry prior to the intervention; the pressure ceiling was set based on published estimates of LES pressure and reflux-inducing negative pressure ranges in the literature. During the expiratory phase, a separate algorithm limits maximum positive pressure to prevent barotrauma. This dual-phase pressure regulation distinguishes the device from conventional resistive breathing trainers, which apply fixed mechanical resistance independent of real-time pressure feedback. The device conceptually targets the crural diaphragm as its primary therapeutic effector through sustained isometric contraction of the inspiratory and expiratory musculature.

Exercise protocol

Training was initiated on Day 1 and performed over 14 consecutive days. The protocol consisted of three sets of 10 repetitions once daily, with three-minute inter-set rest intervals. Each repetition was performed as follows. I placed the mouthpiece of the device to my lips and initiated a slow, controlled inhalation. As inspiratory pressure built, the device’s pressure sensor detected the rising negative pressure and, upon reaching the algorithm-defined threshold, the active valve engaged to restrict further airflow, transitioning me into an isometric inspiratory hold. I sustained this isometric contraction of the diaphragm and accessory inspiratory musculature for two seconds, maintaining effort against the fixed resistance without generating additional airflow. I then transitioned to a controlled exhalation against the device, during which the expiratory safety algorithm similarly limited outward pressure within the permissible range, and an isometric expiratory hold was maintained for an additional two seconds. This constituted one repetition. Scheduled rest days (device not used) were designated on Days 5 and 10. Carbonated beverages were excluded throughout the study period to eliminate exogenous gas intake as a confounding variable. Meal timing and volume were maintained as consistently as practicable. No adverse events, including respiratory discomfort, barotrauma, or worsening of reflux symptoms attributable to device use, were observed during the 14-day intervention period.

Outcome measures

Daily symptoms were assessed twice daily (morning and evening) using a binary-adapted version of the validated six-item GerdQ [7]. The six items were: (1) heartburn, (2) regurgitation, (3) epigastric pain, (4) nausea, (5) sleep disturbance, and (6) rescue medication use. Item 6 was defined as PRN intake of dexlansoprazole 60 mg in the evening due to symptomatic severity (0 = not required; 1 = required). Each item was scored 0 (absent) or 1 (present), yielding a daily maximum binary GerdQ score of 6. This represents a modification of the validated weekly frequency-based scoring to permit day-to-day symptom tracking during the intervention. Morning scores were consistently zero throughout the study and are not separately reported.

Results

Table 1 presents individual item scores and daily totals across the two-day formal baseline and the 14-day intervention period. All morning assessments were 0/6 throughout; only evening scores are presented.

**Table 1:** Daily binary GerdQ evening scores during formal baseline and 14-day intervention period. Each item scored 0 (absent) or 1 (present). Item 6 (Dexlansoprazole PRN): evening rescue intake of dexlansoprazole 60 mg. BL: formal pre-intervention baseline days; †: scheduled rest days (device not used). Total: sum of six items (maximum 6). Morning assessments were 0/6 on all days and are not shown.

Day/Period	Heartburn	Regurgitation	Epigastric Pain	Nausea	Sleep Disturbance	Dexlansoprazole PRN	Total
Baseline Day −2 (BL)	1	1	0	0	1	1	4
Baseline Day −1 (BL)	1	1	0	0	1	1	4
Day 1	0	1	0	0	0	0	1
Day 2	1	1	0	0	0	0	2
Day 3	1	1	0	0	1	1	4
Day 4	0	0	0	0	0	0	0
Day 5†	0	0	0	0	0	0	0
Day 6	1	1	0	0	0	1	3
Day 7	0	0	0	0	0	0	0
Day 8	0	0	0	0	0	0	0
Day 9	1	1	0	0	0	0	2
Day 10†	1	0	0	0	0	0	1
Day 11	0	0	0	0	0	0	0
Day 12	1	1	0	0	0	1	3
Day 13	0	0	0	0	0	0	0
Day 14	0	0	0	0	0	0	0

At baseline (Days −2 and −1), the evening GerdQ score was 4/6 on both days, with heartburn, regurgitation, sleep disturbance, and PRN dexlansoprazole consistently present. Epigastric pain and nausea were absent throughout the entire study.

During the first week of intervention (Days 1-7), daily evening scores ranged from 0 to 4 (mean 1.4 ± 1.6). Symptom-free evenings were observed on Days 4, 5 (rest day), and 7. The highest score of 4 was recorded on Day 3, comprising heartburn, regurgitation, sleep disturbance, and rescue dexlansoprazole. Sleep disturbance was recorded exclusively on Day 3 and was absent for all subsequent days. PRN dexlansoprazole was also required on Day 6 (score 3: heartburn, regurgitation, rescue medication).

During the second week (Days 8-14), daily evening scores ranged from 0 to 3 (mean 0.9 ± 1.1). Symptom-free evenings occurred on Days 8, 11, 13, and 14. PRN dexlansoprazole was required on Day 12 only (score 3: heartburn, regurgitation, rescue medication). A score of 1 (heartburn only) was recorded on the rest day (Day 10), without rescue medication use.

Across the full 14-day intervention period, the mean daily evening score was 1.1 ± 1.4, representing a 71% reduction from the pre-intervention baseline mean of 4.0. Symptom-free evenings comprised 50% of all training days (7/14). PRN dexlansoprazole was required on 3 of 14 days (21%), compared to daily requirement during both the informal observation period and the formal baseline.

## Discussion

This case report describes a 38-year-old physician with more than a decade of PPI-dependent GERD, refractory to multiple prior agents and requiring daily dexlansoprazole 60 mg for symptom control. Following voluntary PPI discontinuation and initiation of a 14-day isometric diaphragmatic resistance training protocol using a novel prototype device, he demonstrated a 71% reduction in the mean daily evening symptom score, resolution of sleep disturbance after a single recurrence on Day 3, symptom-free evenings on 50% of training days, and reduction of rescue PPI use to 3 of 14 days (21%). The degree of symptom reduction and PPI sparing over a short training period is clinically notable given the severity and chronicity of the pre-intervention baseline.

The proposed mechanism centers on progressive crural diaphragm strengthening through isometric respiratory loading. As described above, during inspiration, the generation of negative intrathoracic pressure creates a potential suction gradient across the esophagogastric junction. In patients with a compromised LES, this gradient can facilitate reflux. The crural diaphragm’s inspiratory contraction serves as the principal physiological counterforce against this mechanism, augmenting LES pressure in synchrony with the pressure gradient [4]. Isometric resistive loading of the respiratory musculature may progressively enhance crural diaphragm resting tone and contractile reserve, reducing susceptibility to TLESR-associated inhibition and strengthening the functional esophagogastric junction barrier. The exclusive nocturnal symptom pattern observed in this case, attributable to supine positioning and the loss of gravitational esophageal clearance, places the esophagogastric junction’s intrinsic competence at the center of the pathophysiology, making this patient a particularly well-suited candidate for crural diaphragm-targeted therapy.

This mechanistic rationale is directly supported by the randomized controlled trial of Eherer et al. [6], who demonstrated that structured diaphragmatic breathing exercises significantly reduced esophageal acid exposure time and GERD symptom scores. The present device advances this concept by providing standardized, reproducible, pressure-monitored resistance loading, overcoming the inherent limitations of effort-dependent, uncalibrated conventional breathing techniques. The device’s integrated safety algorithm, which limits inspiratory negative pressure to a threshold below reflux induction, is an important design feature that distinguishes it from uncontrolled breathing exercises and may mitigate the theoretical risk of training-induced reflux exacerbation during high-resistance inspiratory maneuvers.

The recurrent symptomatic episodes on Days 3, 6, 9, and 12 likely reflect the early time course of respiratory muscle adaptation, in which meaningful crural diaphragm hypertrophy and neuromuscular facilitation require sustained progressive training over weeks to months [8]. The observed day-to-day symptom variability is consistent with an early adaptive phase rather than a treatment plateau, suggesting that extended training would yield further and more consistent symptom suppression.

Several limitations merit acknowledgment. This is a single-subject self-experimentation case, precluding causal inference and limiting generalizability. Most critically, the temporal overlap between the intervention period and the expected natural course of rebound acid hypersecretion (RAHS) following long-term PPI discontinuation represents a significant confounding factor that cannot be excluded. Prolonged use of high-dose PPIs is associated with hypergastrinemia-induced parietal cell hyperplasia, and abrupt discontinuation typically results in RAHS that peaks within one to three weeks before gradually attenuating. The formal baseline in this study was recorded approximately seven days after PPI discontinuation, which corresponds to the expected peak of RAHS, while the subsequent 14-day intervention period coincides with the natural attenuation phase. It is therefore not possible to determine whether the observed symptom improvement reflects the effect of the training intervention, the natural resolution of RAHS, or a combination of both. This represents a fundamental limitation of the study design and should be considered when interpreting the results. Placebo effects and regression to the mean similarly cannot be excluded. Furthermore, the corresponding author served simultaneously as the inventor of the device, the sole subject, and the sole outcome assessor, a circumstance that introduces an extreme potential for observer bias and placebo effect that cannot be controlled within a single-subject self-experimentation design. The binary modification of the validated GerdQ from its standard weekly frequency format limits comparability with published normative thresholds, and the use of a modified, unvalidated outcome instrument recorded by an unblinded investigator with significant conflicts of interest further compounds this concern. Objective esophageal measurements-ambulatory pH-impedance monitoring and high-resolution manometry-were not performed, precluding confirmation of reduced TLESR frequency or increased LES pressure. Training parameters were empirically derived and have not been optimized. Future controlled studies should incorporate pH-impedance monitoring, high-resolution manometry, a randomized sham-controlled design, larger cohorts, extended follow-up, and a structured PPI washout period of sufficient duration to allow RAHS resolution prior to baseline assessment.

## Conclusions

This case demonstrates that a 14-day isometric diaphragmatic resistance training protocol using a novel pressure-sensing prototype device was associated with a 71% reduction in evening GERD symptom burden, resolution of sleep disturbance, and reduction of rescue dexlansoprazole use from daily to 3 of 14 days, during the post-PPI discontinuation period in a patient with over a decade of PPI-dependent GERD refractory to multiple prior agents. The exclusively nocturnal symptom pattern implicates TLESR-mediated reflux as the dominant mechanism and identifies crural diaphragm dysfunction as the primary therapeutic target in this case. Controlled trials incorporating pH-impedance monitoring, high-resolution manometry, and an intervention duration of at least four weeks are warranted to distinguish training-specific effects from natural RAHS attenuation following PPI discontinuation.
